# Hippocampal Ripple Coordinates Retrosplenial Inhibitory Neurons during Slow-Wave Sleep

**DOI:** 10.1016/j.celrep.2019.12.038

**Published:** 2020-01-14

**Authors:** Ashley N. Opalka, Wen-qiang Huang, Jun Liu, Hualou Liang, Dong V. Wang

**Affiliations:** 1 Department of Neurobiology and Anatomy, Drexel University College of Medicine, Philadelphia, PA 19129, USA; 2 School of Biomedical Engineering, Drexel University, Philadelphia, PA 19104, USA; 3 Lead Contact

## Abstract

The hippocampus and retrosplenial cortex (RSC) play indispensable roles in memory formation, and importantly, a hippocampal oscillation known as ripple is key to consolidation of new memories. However, it remains unclear how the hippocampus and RSC communicate and the role of ripple oscillation in coordinating the activity between these two brain regions. Here, we record from the dorsal hippocampus and RSC simultaneously in freely behaving mice during sleep and reveal that the RSC displays a pre-ripple activation associated with slow and fast oscillations. Immediately after ripples, a subpopulation of RSC putative inhibitory neurons increases firing activity, while most RSC putative excitatory neurons decrease activity. Consistently, optogenetic stimulation of this hippocampus-RSC pathway activates and suppresses RSC putative inhibitory and excitatory neurons, respectively. These results suggest that the dorsal hippocampus mainly inhibits RSC activity via its direct innervation of RSC inhibitory neurons, which overshadows the RSC in supporting learning and memory functions.

## In Brief

Converging evidence suggests that hippocampal ripple oscillations and their interaction with the neocortex are critical for memory consolidation. By combining electrophysiology and optogenetic techniques in freely behaving mice, Opalka et al. provide direct evidence that hippocampal ripples communicate with retrosplenial cortex (RSC) interneurons and inhibit RSC population activity during sleep-associated memory consolidation.

## Graphical Abstract

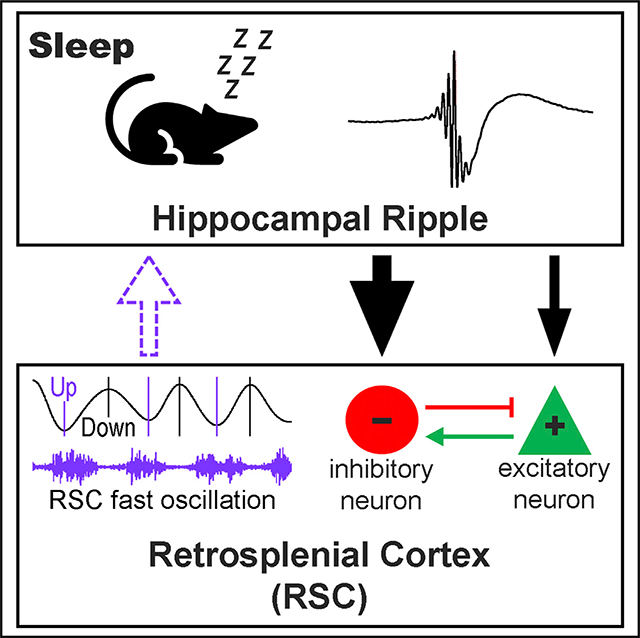

## INTRODUCTION

Sleep promotes memory consolidation, a process that gradually transforms newly acquired information into long-term memory, which takes days, weeks, or even longer (Squire, 1992; Walker and Stickgold, 2004; Diekelmann and Born, 2010; Kitamura et al., 2017). In particular, slow-wave sleep (SWS), characterized by large-amplitude slow oscillations, has been shown to be critical during the memory consolidation process (Marshall et al., 2006; Rasch et al., 2007; Peigneux et al., 2004). Growing evidence suggests that consolidation of a memory involves a large neural network of multiple brain regions, including the hippocampus and retrosplenial cortex (RSC) (Squire and Alvarez, 1995; Nadel and Moscovitch, 1997; Cowansage et al., 2014; Todd and Bucci, 2015; Kitamura et al., 2017; de Sousa et al., 2019; Yamawaki et al., 2019). However, the interaction and information exchange between these brain regions during the memory consolidation process remain unclear.

Much progress has been made in recognizing the hippocampus and its interaction with neocortical regions as crucial for memory consolidation (Squire and Alvarez, 1995; Nadel and Moscovitch, 1997). In particular, a hippocampal sharp-wave ripple, a 150- to 250-Hz oscillation that occurs predominantly during SWS and awake immobility, has been shown to play a key role in memory consolidation (Girardeau et al., 2009; Ego-Stengel and Wilson, 2010; Jadhav et al., 2012; Wang et al., 2015; Ylinen et al., 1995; Buzsáki, 2015). Coinciding with ripples, hippocampal neural ensembles display activity patterns that are reminiscent of prior experience-related ensemble patterns, a phenomenon known as “memory replay” (Wilson and McNaughton, 1994; Skaggs and McNaughton, 1996). This ripple-associated memory replay potentiates relevant synaptic connections and transforms hippocampus-dependent memory into cortex-dependent memory. However, direct evidence that supports this hypothesis of hippocampus-to-cortex information transduction for memory consolidation is limited (Wierzynski et al., 2009; Nir et al., 2011; Todorova and Zugaro, 2019).

Recent studies have provided evidence that the RSC plays a critical role in the acquisition and likely consolidation of new memories (Keene and Bucci, 2008; Katche et al., 2013; Kwapis et al., 2015; Todd and Bucci, 2015; de Sousa et al., 2019; Cowansage et al., 2014; Yamawaki et al., 2019). The RSC also stands out as one of few neocortical regions that receives direct input from the dorsal hippocampus (van Groen and Wyss, 1990; Wyss and Van Groen, 1992), positioning the RSC as a potential bridge for hippocampus-neocortex dialog. However, how the hippocampus communicates with the RSC has come under scrutiny only recently. An earlier study that performed multisite recording across many cortical regions reported ripple-correlated activity in the RSC (Battaglia et al., 2004). Two later studies reported a coordinated theta/gamma coupling, along with ripple-related interactions, between the hippocampus and RSC during awake state and rapid-eye movement sleep (Koike et al., 2017; Alexander et al., 2018). Utilizing electrophysiology and optogenetic approaches in freely behaving mice, this current study aims to determine how hippocampal ripples interact with the RSC during SWS for potential consolidation of memories, and how neural oscillations facilitate the interaction between the two brain regions.

## RESULTS

### Coordinated Hippocampal Ripples and RSC Oscillations during SWS

**Figure 1.**
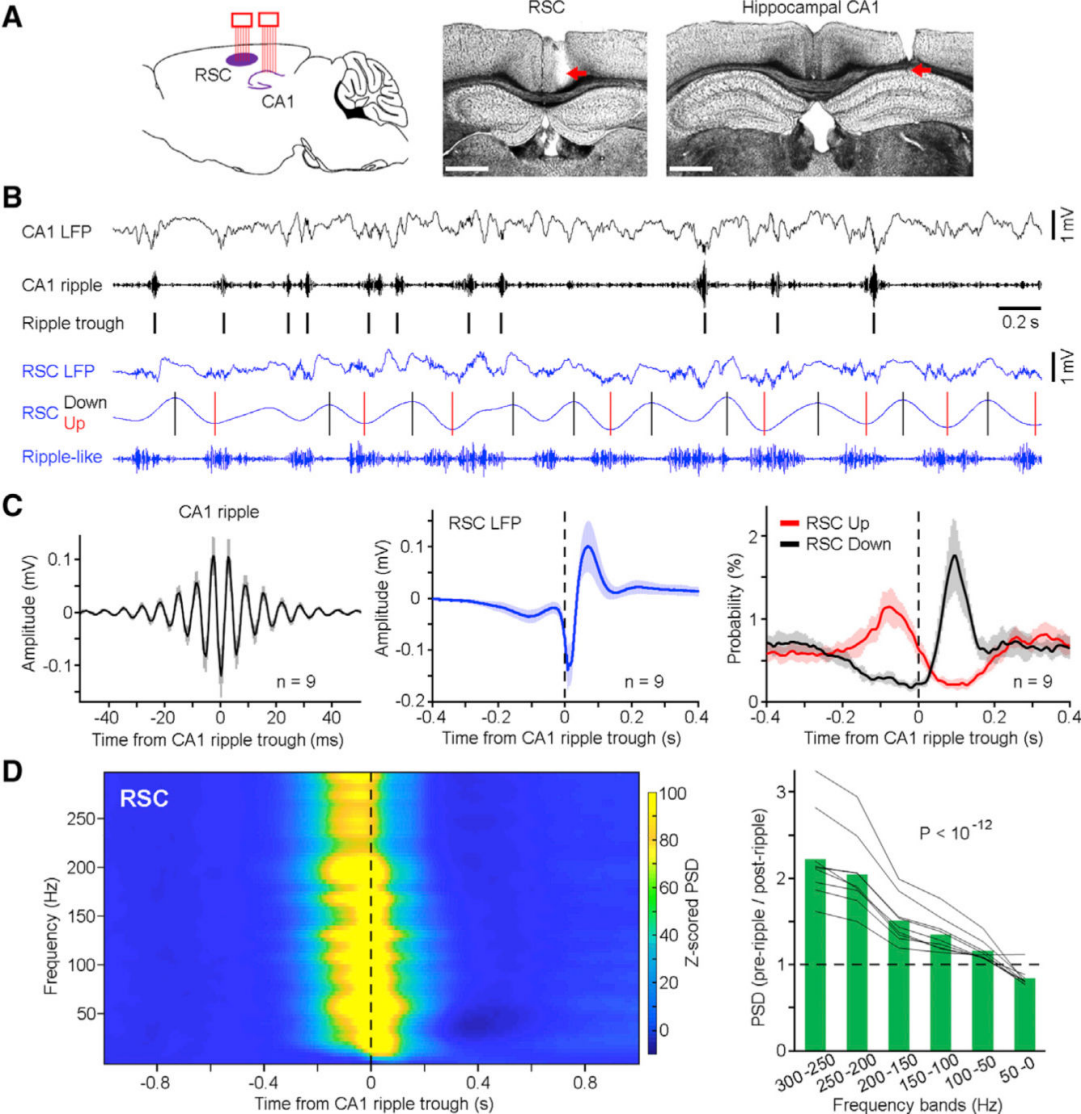
Coordinated Hippocampal Ripples and RSC Oscillations (A) Left, schematic drawing of dual-site multitetrode recording in the hippocampal CA1 and RSC. Two bundles of tetrodes (eight each) were implanted into the two regions. Middle and right, representative coronal sections showing recording sites in the granular RSC and hippocampal CA1, respectively. Scale bars, 0.1 mm. (B) Representative simultaneously recorded CA1 and RSC LFPs (black and blue, respectively) and band-pass filtered oscillations (ripple: 150–250 Hz; up/down: 1–4 Hz, where black and red bars indicate peaks and troughs, respectively. Both were used for analyses in C, right). (C) Left and middle, mean CA1 ripples ± SD and mean RSC LFPs ± SD, respectively, in relation to CA1 ripple trough (n = 9 mice). Right, mean probability of RSC Up/Down states ± SD in relation to CA1 ripple trough (n = 9 mice). SD, standard deviation. (D) Left, mean peri-ripple spectrogram of RSC LFPs in relation to CA1 ripple trough (n = 9 mice). PSD, power spectral density. *Z* score transform was based on mean and SD calculated between − 1 and −0.5 s (see STAR Methods). Right, ratio of pre-ripple (−0.2–0 s) to post-ripple (0−0.2 s) RSC PSD at each frequency band. Green bars indicate the mean; gray lines indicate individual mice (n = 9). One-way ANOVA revealed a decrease of ratio across frequency bands (F(_5,40_) = 26.58, p< 10^−12^). See also Figures S1 and S2.

To determine how the hippocampus and RSC interact during information exchange, we implanted two bundles of tetrodes into the hippocampal CA1 and ipsilateral granular RSC (Figure 1A; see STAR Methods). Neural activity recorded from a total of nine mice was used for analyses (Figure S1, left). We first analyzed CA1 and RSC local field potentials (LFPs) during SWS–a brain state critical for memory consolidation (Diekelmann and Born, 2010; Walker and Stickgold, 2004). Our results revealed that both the CA1 and RSC exhibited high-frequency oscillations (rippleand ripple-like, respectively), with the RSC ripple-like oscillation occurring concurrently with RSC Up states (Figure 1B). Next, we identified individual ripple events (Figure 1C, left) and then used each ripple event as a reference to examine RSC activity. Our analyses revealed that the RSC displayed a small-amplitude depolarization (~100-ms pre-ripple), followed by a sharp depolarization (0–20 ms) and then a rebound (20–100 ms) hyperpolarization (Figure 1C, middle). In addition, the RSC Up and Down states tended to occur pre- and post-ripple, respectively (Figure 1C, right), indicating a high probability of CA1 ripple occurrence at the RSC Up-to-Down transition. These results provided evidence that there is coordinated activity between the hippocampus and RSC during SWS.

To further examine a broad band of RSC oscillations in relation to CA1 ripple, we conducted a peri-ripple spectrogram analysis of RSC LFP. Across the fast-to-slow oscillation bands (300-to-0 Hz), there is a shift of RSC oscillation power from pre- to post-ripple during SWS (Figures 1D and S2). This shift of oscillation power was consistent across all animals (Figure 1D, right) with no obvious differences among subregions of the granular RSC and, thus, may serve as a signature of hippocampal ripple-RSC interaction. To determine whether this shift was a general phenomenon, we also conducted dual-site recordings of the CA1 and several other brain regions, including the anterior cingulate cortex (ACC), median raphe (MnR), or diagonal band of Broca (DBB). Our analyses did not reveal a shift of oscillation power in any of these three brain regions in relation to hippocampal ripple during SWS (Figure S1).

### RSC Excitatory and Inhibitory Neurons Are Reciprocally Connected

**Figure 2.**
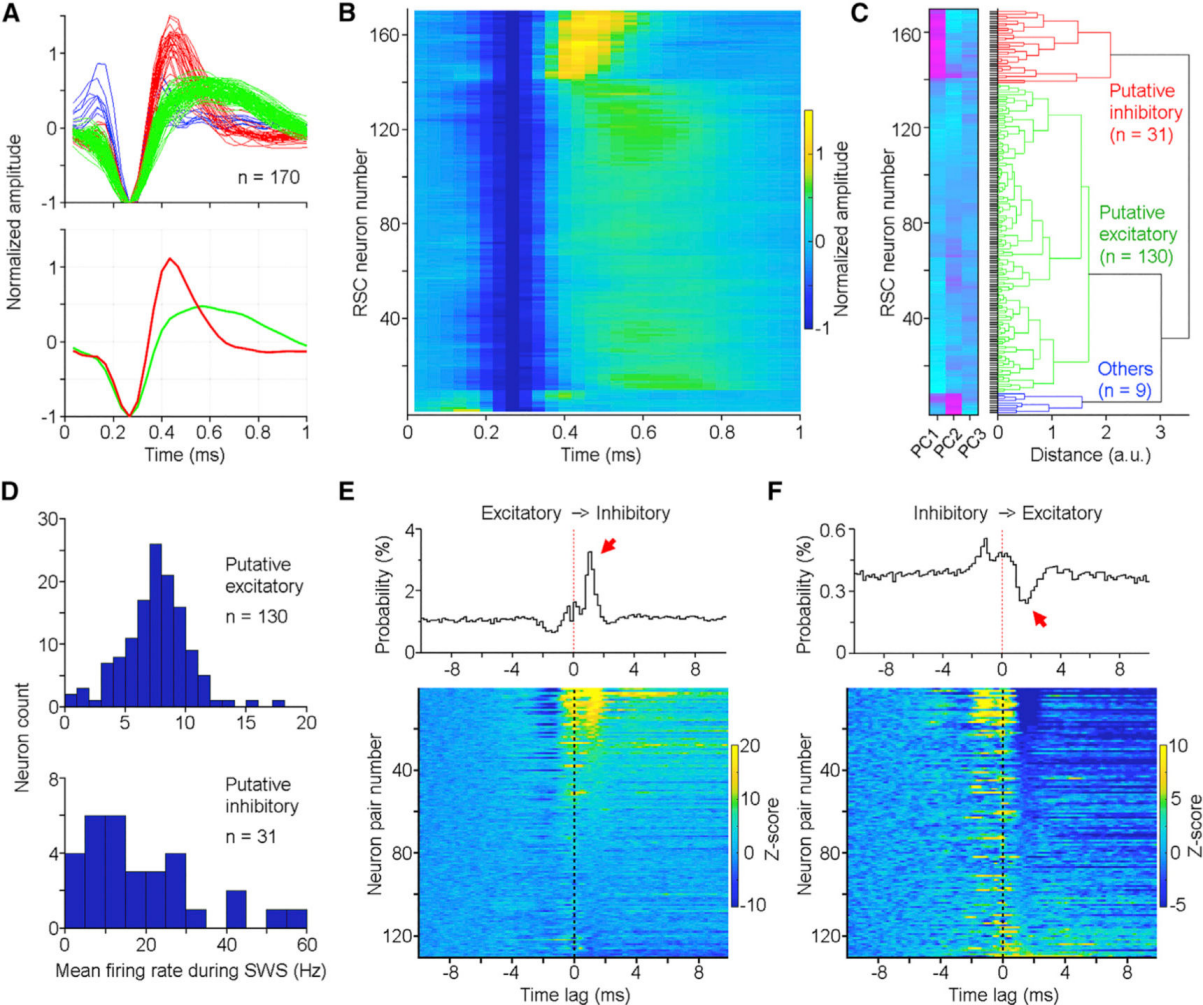
RSC Putative Excitatory and Inhibitory Neurons (A) Normalized spike waveforms of RSC neurons individually (top; n = 170) and averaged (bottom). Red, green, and blue correspond to putative inhibitory, excitatory, and other neurons, respectively. (B) Spike waveform heatmap of the same 170 RSC neurons as shown in (A). (C) Principal-component analysis(PCA) classifies RSC neurons into putative inhibitory (red; n = 31), excitatory (green; n = 130), and others (blue; n = 9). Neurons in (B) and (C) are arranged in the same order. PC1, PC2, and PC3 represent the first three principal components color coded from low (turquoise) to high scores (magenta). (D) Mean firing rate distribution of RSC putative excitatory (top) and inhibitory (bottom) neurons during SWS. (E and F) Cross-correlations between RSC putative excitatory and inhibitory neurons. Top panels, cross-correlation histograms of two representative neuron pairs; bottom panels, cross-correlation histogram heatmaps of individual neuron pairs (n = 130 pairs; only neuron pairs recorded from different tetrodes were used in this analysis; inhibitory neurons were used as the reference in E, and excitatory neurons were used as the reference in F). Color bars indicate z-scored firing probability, and neurons are arranged from high-to-low firing in (E) and low-to-high firing in (F) based on a time window of 1−2 ms. *Z* score transform was based on mean and SD calculated between −10 and −5 ms. See also Figure S3.

Like other cortical regions, the RSC contains both excitatory and inhibitory neurons (Salaj et al., 2015). To determine how different types of RSC neurons are engaged in neural oscillations, one of the first steps was to classify our recorded RSC neurons into subtypes. Previous classifications of neuron types often reported distinct spike waveforms and/or activity patterns between excitatory and inhibitory neurons. Here, we classified RSC neurons based on spike waveforms using an unsupervised method–the principal-component analysis (PCA). Our PCA classified the RSC neurons into three major categories (Figures 2A–2C): putative excitatory neurons (green; 130/170), putative inhibitory neurons (red; 31/170), and others (blue; 9/170). For the following analyses, we focused on the first two categories due to the small number in the third category.

Consistent with previous established criteria, these RSC putative excitatory neurons exhibited wider spike widths compared to putative inhibitory neurons (Figure 2A). In addition, most of the classified putative excitatory neurons exhibited a lower firing rate (3–12 Hz; 120 out of 130) compared to many putative inhibitory neurons (Figure 2D, bottom). A further cross-correlation analysis revealed that these two neuron types tended to excite and inhibit each other, respectively, with a latency of 2 ms or shorter (Figures 2E and 2F), further validating their excitatory and inhibitory identities (English et al., 2017). These results also suggest that the RSC putative excitatory and inhibitory neurons are reciprocally connected because cross-correlation histograms often exhibit a double-phase correlation, that is, an inhibition (between −2and −1 ms) followed by activation (between 1 and 2 ms) or vice versa (Figures 2E and 2F). On the other hand, RSC putative excitatory neurons rarely excited each other; rather, a subset displayed synchronized activity within ±1 ms Figure S3), indicating a shared common input to RSC excitatory neurons.

### Hippocampal Ripple-Correlated RSC Activity

**Figure 3.**
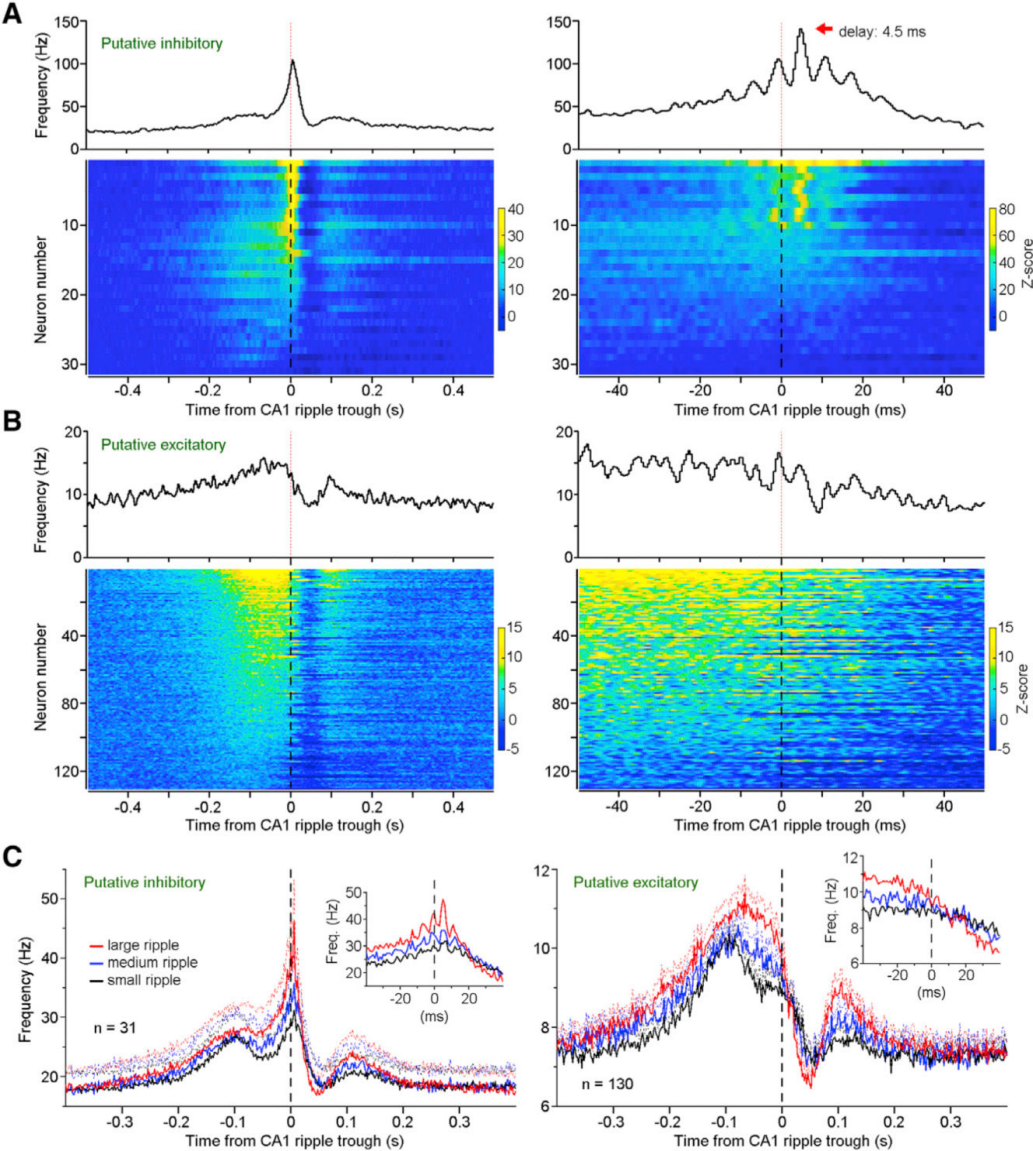
Hippocampal Ripple-Correlated RSC Activity (A and B) Top panels, mean frequency of one RSC putative inhibitory neuron (A) and one RSC putative excitatory neuron (B); bottom panels, heatmap of all RSC putative inhibitory (A) (n = 31) and excitatory neurons (B) (n = 130) in relation to CA1 ripple trough. Neurons in the left and right panels are the same neurons arranged in the same order (bin = 2 and 0.5 ms, respectively). *Z* score transform was based on mean and SD calculated between −1and −0.5 s. (C) Mean frequencies (solid lines) and SEM (dashed lines) of RSC putative inhibitory (left; n = 31) and excitatory neurons (right; n = 130) in relation to different sizes of ripple events (red, large; blue, medium; black, small). See also Figures S2 and S4.

Our analyses of RSC LFPs revealed that the RSC displayed a sharp post-ripple depolarization (Figure 1C, middle). To further characterize this hippocampus-to-RSC latency, we conducted cross-correlations analyses between hippocampal ripples and the identified RSC putative inhibitory and excitatory neurons. These analyses revealed that most RSC putative inhibitory neurons (87.1%, 27/31) displayed a post-ripple firing increase (0−10 ms; *Z* score > 3.3; see STAR Methods; Figure 3A, left). Importantly, a subset (32.3%, 10/31) displayed a clear phase locking relative to CA1 ripple, with a delay of 4.6 ± 0.9 ms (mean ± SD; ranged between 3.5 and 6.0 ms; Figure 3A, right). This short delay (4.6 ms) indicates a monosynaptic hippocampus-to-RSC connection, given that hippocampal pyramidal neurons in both the CA1 (Figure S3) and subiculum (Chrobak and Buzsáki, 1996) fire at ripple trough. Conversely, RSC putative excitatory neurons displayed little phase locking relative to ripples and, instead, decreased firing (~50 ms post-ripple) followed by a rebound activation (~100 ms post-ripple; Figure 3B). While it is possible that hippocampal ripples can directly activate some RSC excitatory neurons, this excitatory effect seems to be smaller and less probable than activation of RSC inhibitory neurons. Therefore, we speculate that hippocampal ripples mainly suppress RSC activity through a direct activation of RSC inhibitory neurons.

On the other hand, our analyses also revealed a pre-ripple activation of RSC putative inhibitory and excitatory neurons during SWS (Figures 3A and 3B; further analyses using ripple onset as the reference reached the same conclusion; see Figure S2). To determine how closely the pre- and post-ripple RSC activations correlated with hippocampal ripple size, we classified ripple events into three categories based on ripple amplitude: large, medium, and small (see STAR Methods). Our analysis revealed that the post-ripple firing rate of RSC putative inhibitory neurons (averaged between 0 and 10 ms) was proportional to ripple size (F(_2,60_) = 31.71, p < 10^−9^, repeated-measures ANOVA; Figure 3C, left). In addition, the pre-ripple activity of both putative inhibitory and excitatory neurons in the RSC (averaged between −100 and 0 ms) was proportional to ripple size (F(_2,60)_ = 32.54, p < 10^−9^, and F(_2,258)_ = 110.28, p < 10^−34^, respectively, repeated-measures ANOVA; Figure 3C), indicating a potential RSC influence on hippocampal ripple and related reactivation contents (Ji and Wilson, 2007; Rothschild et al., 2017).

In addition, we analyzed hippocampal ripple-associated RSC activity during awake immobility (ripples were recorded mostly during food consumption). Unlike the SWS state, there was little pre-ripple depolarization or ripple-like oscillation in the RSC during awake immobile, particularly between −200 and −100 ms (Figure S4A). Similarly, there was little pre-ripple firing change of RSC putative inhibitory or excitatory neurons during awake immobility (Figures S4B–S4D). Conversely, most RSC putative inhibitory neurons displayed an activation after ripple during awake immobility, similarly seen during SWS state (Figures S4C and S4E).

### A Monosynaptic Hippocampus-to-RSC Connection

**Figure 4.**
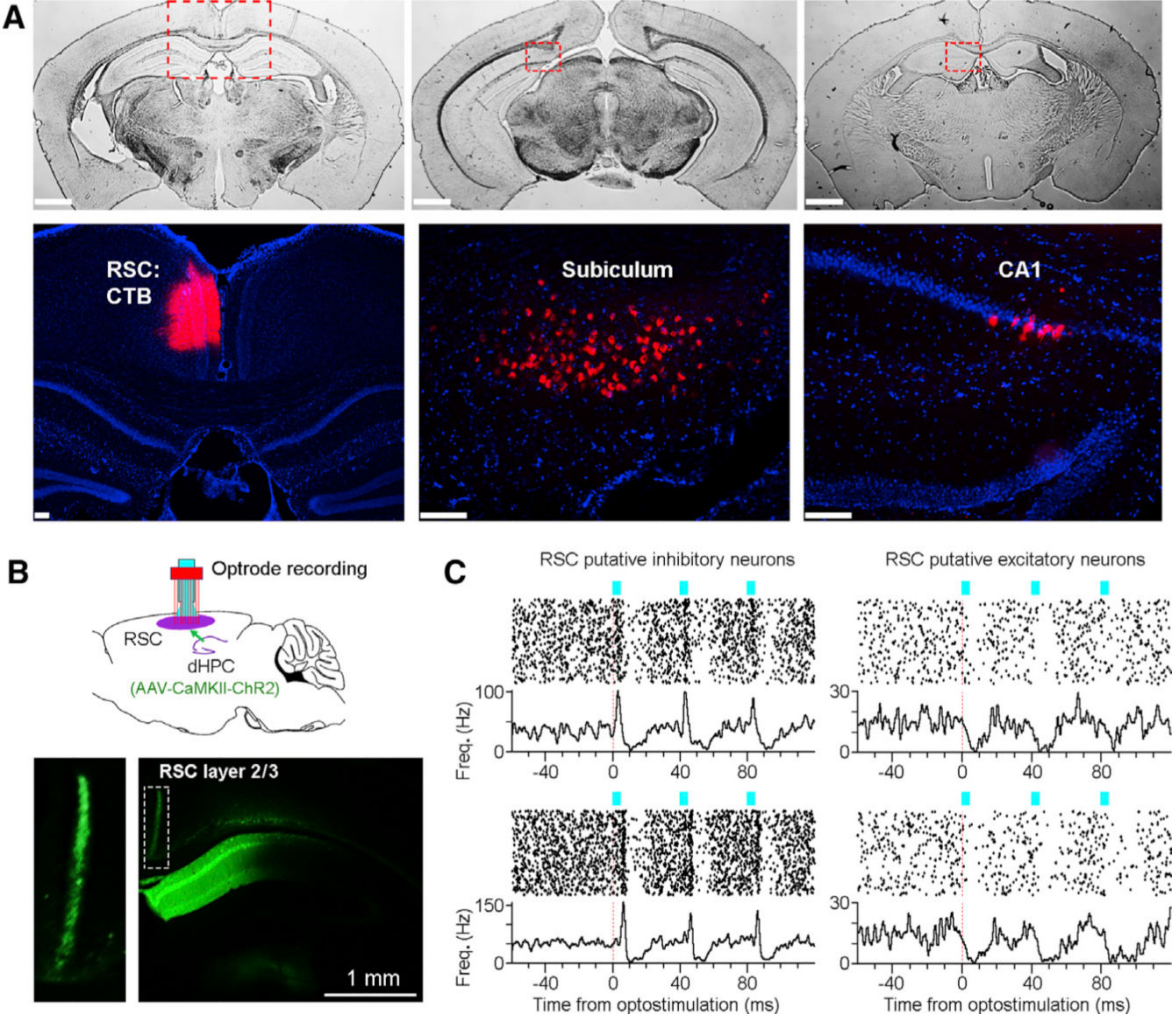
A Dorsal Hippocampus-to-RSC Connection (A) Top panels, three coronal sections (from the same brain) showing CTB injection in the RSC (left) and retrograde labeling of CTB densely in the dorsal subiculum (middle) but sparsely in the rostral medial CA1 (right). Bottom panels, zoomed- in of the dashed squares in top panels; red indicates CTB; blue indicates DAPI staining. Scale bars, 1 mm (top) and 0.1 mm (bottom). (B) Top, schematic drawing of RSC optrode recording (an optical fiber attached to eight tetrodes). AAV1-CaMKII-ChR2-GFP virus (0.2 pL) was injected into the medial dorsal CA1 (the virus also spread to the dorsal subiculum; see STAR Methods). Bottom, a representative coronal section showing hippocampal/subicular efferents in granular RSC layer 2/3 (inset). (C) Optostimulation ofthe dorsal hippocampus-to- RSC terminals (three pulses at 25 Hz; pulse width, 3 ms; laser power, 1 mW; n = 292 trials) activated RSC putative inhibitory neurons (left) and suppressed putative excitatory neurons (right). The four RSC neurons were recorded simultaneously. See also Figure S5.

To determine which hippocampal subregions project directly to the RSC, we injected cholera toxin subunit B (CTB) retrograde tracers into the RSC. Our results revealed that the RSC receives a dense projection from the dorsal subiculum (Figure 4A). In addition, the RSC also receives a direct projection, albeit being relatively weak, from the rostral medial CA1 and fasciola cinerea regions (Figures 4A and S5).To confirm that these dorsal hippocampal projections can directly activate RSC neurons, we utilized a combined optogenetic and electrophysiology approach (Figure 4B): we injected AAV-CaMKII-ChR2 viruses (0.2 μL) into the dorsal hippocampus (which spread to the medial CA1, fasciola cinerea, and dorsal subiculum regions) and implanted an optrode (an optical fiber attached to eight tetrodes) into the ipsilateral granular RSC. The use of the CaMKII promoter likely enabled the expression of ChR2 selectively on hippocampal pyramidal (Wang et al., 2013; Zhu et al., 2014; Stark et al., 2012), although we cannot rule out the possibility of a potential ChR2 expression on interneurons.

**Figure 5.**
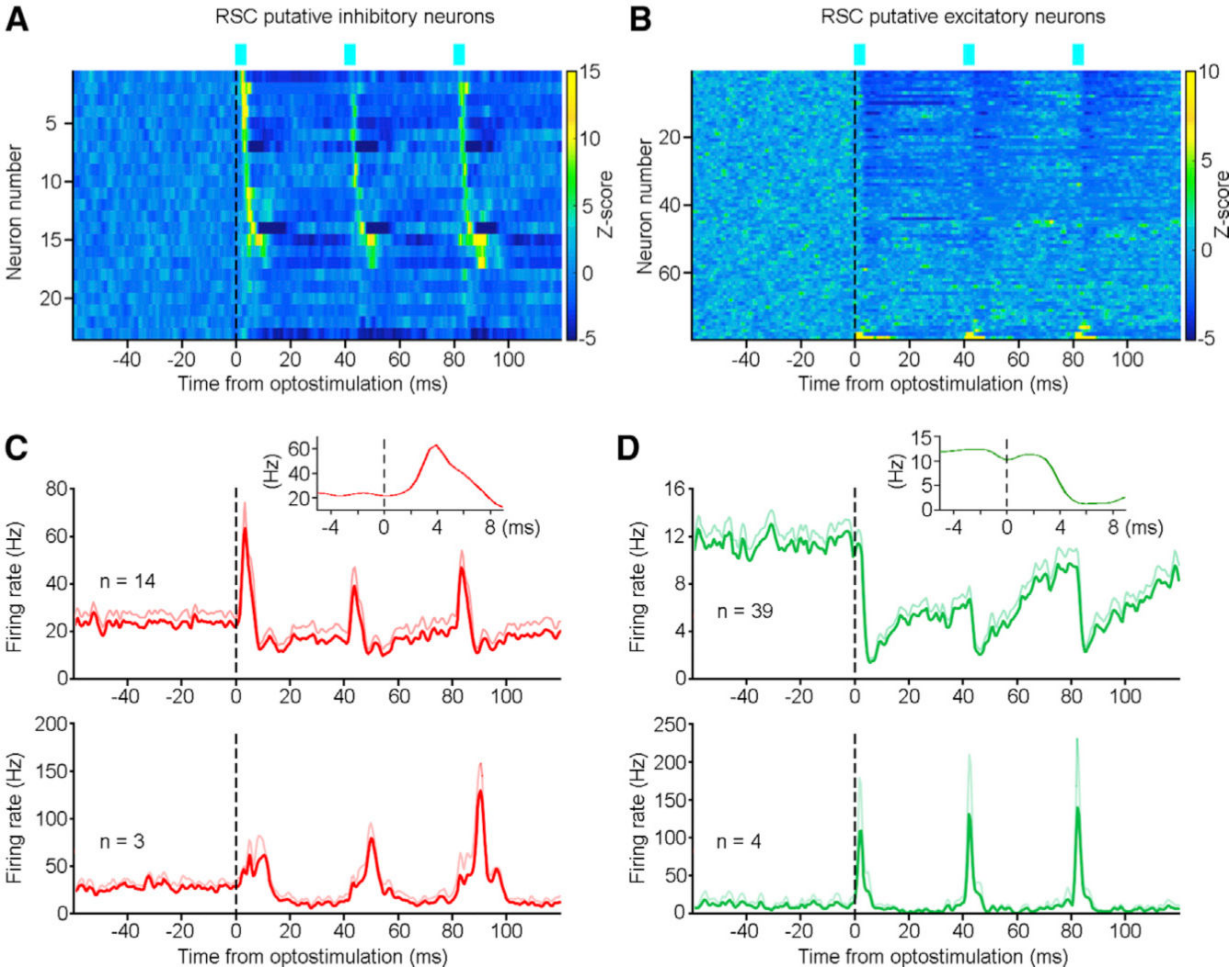
A Monosynaptic Hippocampus-to-RSC Innervation (A and B) Activity heatmap of RSC putative inhibitory (A) (n = 23) and excitatory (B) (n = 79) neurons upon optostimulation of the dorsal hippocampus-to-RSC terminals. Color bars indicate z-scored firing probability; z-score transform was based on mean and SD calculated between −60 and 0 ms. (C) Mean activity (red lines) and SEM (light red lines) of fast-response (top; n = 14) and slow-response (bottom; n = 3) RSC putative inhibitory neurons upon optostimulation. The fast- and slow- response latencies ranged between 2.5−6 and 9.5−10 ms, respectively. Inset, mean firing of the fast-response RSC neurons (n = 14) peaked at ~4 ms upon optostimulation. (D) Mean activity (green lines) and SEM (light green lines) of inhibited (top; n = 39) and activated (bottom; n = 4) RSC putative excitatory neurons upon optostimulation. Inset, mean firing of the inhibited RSC neurons (n = 39) troughed at ~6 ms upon optostimulation. Laser: three pulses at 25 Hz; pulse width, 3 ms; power, ~1 mW. See also Figure S5.

Our results revealed that optostimulation of the hippocampal projections in the RSC activated and inhibited RSC putative inhibitory and excitatory neurons, respectively (Figure 4C). Overall, most RSC putative inhibitory neurons were activated upon optostimulation (73.9%, 17/23; *Z* score > 3.3, see STAR Methods; Figures 5A and 5C). The response latency–defined as latency to the peak firing rate–for most RSC putative inhibitory neurons was short (3.9 ± 1.0 ms, mean ± SD; ranged between 2.5 and 6 ms; n = 14), indicating a monosynaptic hippocampus-to-RSC connection (Figure 5C, top). Additionally, a few RSC putative inhibitory neurons (n = 3) had a response latency of 9.5−10 ms, likely polysynaptic (Figure 5C, bottom).

In contrast, many simultaneously recorded RSC putative excitatory neurons were suppressed (49.4%, 39/79; *Z* score less than −3.3; see STAR Methods) upon the same optostimulation (Figures 5B and 5D). Only a small fraction of RSC putative excitatory neurons was activated (5.1%, 4/79; response latency, 3.1 ± 1.1 ms; Figure 5D, bottom), indicating a relatively weak projection from the hippocampus to the RSC excitatory neurons, or that this excitatory effect was overrode by a strong feedforward inhibition (Jackson et al., 2018) via RSC inhibitory neurons. These results, corroborated with our dual-site recording data (Figure 3), indicating a monosynaptic innervation from the dorsal hippocampus to the RSC, predominantly targeting RSC inhibitory neurons.

### Clusters of RSC Activity Co-terminate with Ripples

**Figure 6.**
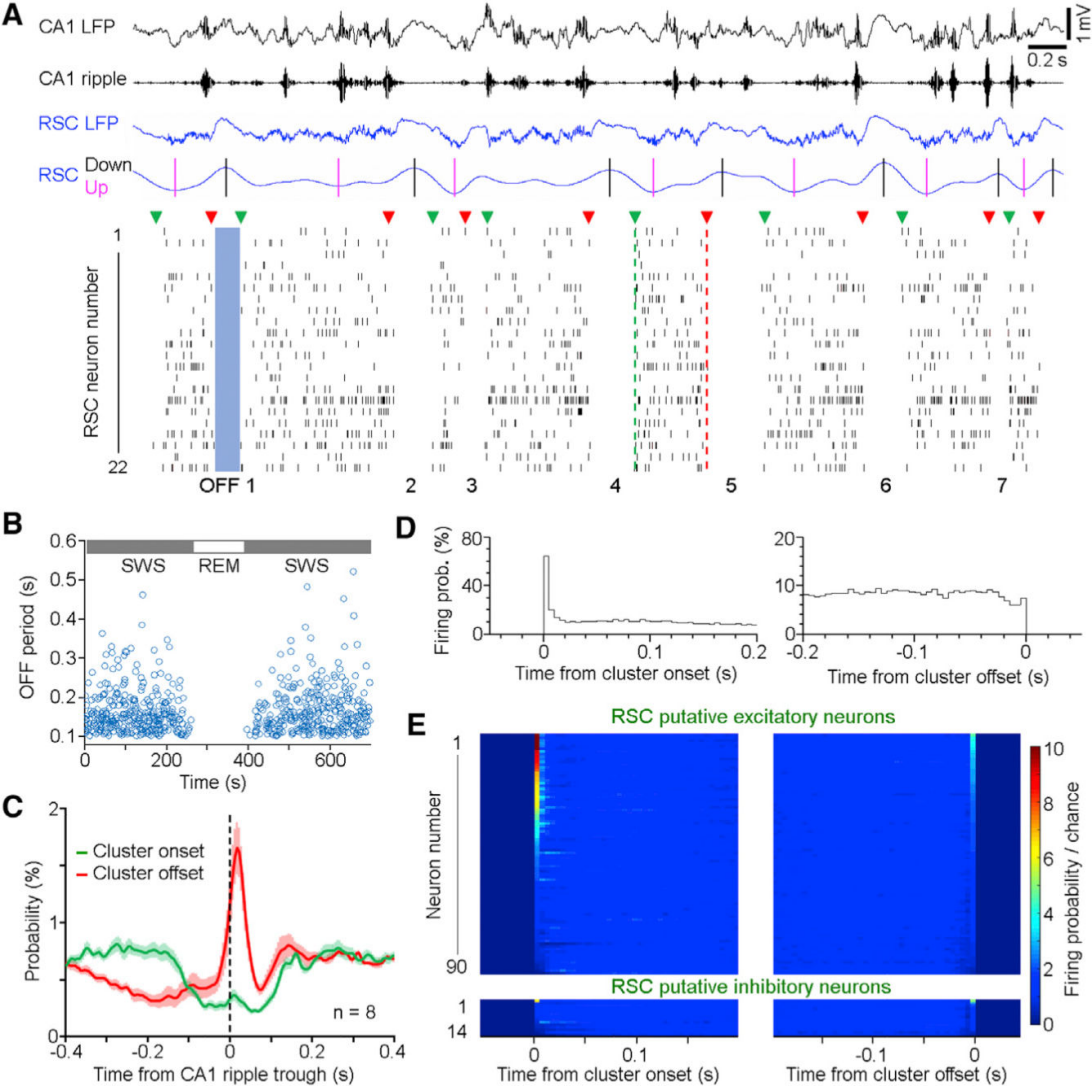
Clusters of RSC Activity Co-terminate with Hippocampal Ripple (A) Representative simultaneously recorded CA1/RSC oscillations (top; black/blue, respectively; up/ down: 1–4 Hz, where black and magenta bars indicate peaks and troughs, respectively) and spikes of 22 RSC neurons (bottom; neurons 4, 15, and 16 are putative inhibitory, whereas the rest are putative excitatory neurons). RSC neurons fired in clusters with an “ON-OFF” cyclic pattern: green triangles indicate cluster onsets; red triangles indicate cluster offsets. An OFF period is defined by none of the RSC neurons firing within a period of 0.1 s or longer. (B) Durations of OFF periods (blue circles) during SWS and REM sleep. No OFF period was detected during REM. (C) Mean probability ± SEM of RSC cluster onsets (green) and offsets (red) in relation to CA1 ripple trough. (D) Firing probability of one RSC putative excitatory neuron in relation to the onset (left) and offset (right) of RSC clusters. (E) Normalized firing probability of RSC putative excitatory (top; n = 90) and inhibitory neurons (bottom; n = 14) in relation to RSC cluster onsets/offsets. Chance was defined by shuffled spike firing probability. Neurons are arranged from high to low firing based on the time window of 0−5 ms. See also Figure S6.

Next, we asked how RSC population activity may correlate with CA1 ripples during SWS. By examining raw spike sequences, it was noticeable that the RSC neurons fired in clusters with “ON” and “OFF” cyclic patterns, which correlates with up and down states of RSC slow waves (Figure 6A; see STAR Methods). Remarkably, during the OFF period, often no single RSC spike fired (Figure 6A). Therefore, we defined the OFF period as no RSC spike firing within a duration of 100 ms or longer for further analysis, which only included data with eight or more RSC neurons recorded simultaneously. This analysis showed that the OFF period was short-lasting (<600 ms) and only occurred during SWS, but not awake state or rapid-eye movement (REM) sleep (Figure 6B), which is consistent with a recent study (Koike et al., 2017).

To further characterize these RSC clusters, we identified the onset and offset of each cluster, which corresponded to the first and last spikes within each cluster, respectively (Figure 6A; green and red triangles). Our analyses revealed that the RSC cluster activity had a high probability of termination immediately after ripples (Figure 6C, left). This suggests that hippocampal ripples could contribute to the termination of RSC population activity, or that hippocampal ripples and RSC population activity co-terminate via a general thalamocortical regulation of Up and Down states (Crunelli et al., 2015). Given that CA1 ripples also tend to occur in clusters (Davidson et al., 2009), we further analyzed RSC activity in relation to CA1 ripple cluster onsets and offsets. These analyses revealed subtle differences of ripple-correlated RSC activity between the two time points (Figure S6). Notably, RSC cluster of spikes and the CA1 cluster of ripples tended to co-terminate (Figure S6).

It is plausible that the RSC cluster activity could represent coding units of information. Therefore, we sought to determine whether any reoccurring patterns of activity existed across RSC clusters. Indeed, a subset of RSC putative excitatory neurons (30/90) tended to fire at the cluster onset, displaying a high probability (defined here as 5-fold or higher above chance; Figures 6D, left, and 6E, top left). This indicates the existence of patterned activity across RSC clusters. Conversely, many RSC putative excitatory neurons fired close to chance level at the cluster offset, while none of the same neurons had a comparably high firing probability at cluster offsets (Figures 6D, right, and 6E, top right). On the other hand, very few RSC putative inhibitory neurons (only 1/14) displayed high firing probability at RSC cluster onsets/offsets (Figure 6E, bottom panels). These above analyses indicate a recurring pattern of RSC population activity that could potentially communicate with hippocampal ripples during SWS.

## DISCUSSION

Here, we reported coordinated activity between the RSC and hippocampal ripples during SWS: a pre-ripple general activation and a post-ripple selective activation in the RSC. Immediately after ripples, a subset of RSC putative inhibitory neurons increased activity, while RSC putative excitatory neurons largely decreased activity. Consistently, optogenetic stimulation of the hippocampus-to-RSC pathway activated and inhibited RSC putative inhibitory and excitatory neurons, respectively. These results indicate a potential two-way communication between the hippocampus and RSC: the hippocampus mainly inhibits RSC activity through direct activation of RSC inhibitory neurons, while the RSC may influence hippocampal ripples through coordinated slow and fast oscillations.

Neural oscillations have long been perceived as important for coordinating neural activity among brain regions. In particular, slow waves (up-down states) are thought to be generated by the thalamocortical network, likely playing a key role in the coordination of various cortical regions (Crunelli et al., 2015). Fast oscillations (>100 Hz), while well studied in the hippocampus, have also been observed in the cortex recently; however, less is known about the physiological mechanisms and functional significance (Buzsáki and Silva, 2012; Averkin et al., 2016; Kucewicz et al., 2014). The current study is consistent with recent observations of ripple-like fast oscillations in association cortices, including the RSC (Khodagholy et al., 2017). These RSC fast oscillations occurred predominantly during RSC Up state and were coupled with hippocampal ripples, indicating a coordinated hippocampus-RSC oscillation network.

### A Hippocampus-to-RSC Influence

Our dual-site *in vivo* recording revealed that a subpopulation of RSC putative inhibitory neurons increased firing phase-locked to hippocampal ripple rhythm after a delay of 4.6 ms, indicating a monosynaptic projection from the dorsal hippocampus to RSC inhibitory neurons. This is consistent with anatomical evidence that showed a direct projection from the dorsal hippocampus, primarily from the dorsal subiculum (Wyss and Van Groen, 1992; Yamawaki et al., 2019) and secondarily from the medial CA1, both glutamatergic (Haugland et al., 2019; van Groen and Wyss, 1990) and GABAergic (Jinno et al., 2007; Miyashita and Rockland, 2007), to the granular RSC layer 2/3. Our CTB retrograde labeling corroborated with these earlier findings and provided evidence that the rostral medial CA1 and fasciola cinerea regions also project directly to the RSC. Therefore, both the dorsal subiculum and medial CA1 could drive the firing of RSC inhibitory neurons during ripple oscillation, given that both the subiculum (Chrobak and Buzsáki, 1996) and CA1 neurons (Figure S3) fire phase-locked to CA1 ripple rhythm. On the other hand, the RSC putative excitatory neurons largely decreased firing after hippocampal ripples, likely through an inhibition from local interneurons (Alexander et al., 2018). Notably, RSC putative inhibitory and excitatory neurons influenced each other’s activity (Figures 2E and 2F), regardless of the presence of hippocampal ripples.

Our additional optogenetic stimulation confirmed that the dorsal hippocampus efferents mainly activated RSC putative inhibitory neurons, while inhibiting putative excitatory neurons, albeit a non-physiological nature of the stimulation due to an extremely high synchrony. Most putative inhibitory neurons were likely activated monosynaptically (with a delay of 2.5−6 ms), whereas a small subset seemed to be activated polysynaptically (with a delay of 9.5−10 ms). Given that our adenoassociated virus (AAV) injection (0.2 μL) infected both the medial CA1 and dorsal subiculum, and that the RSC receives more inputs from the subiculum than CA1, the excitatory effect on RSC putative inhibitory neurons should be attributed primarily to the subiculum inputs and, secondarily, the CA1 inputs. On the other hand, hippocampal projections mainly suppressed RSC putative excitatory neurons, potentially mediated by a feedforward inhibition through RSC interneurons.

Notably, a small percentage (~5%) of RSC putative excitatory neurons was strongly activated upon optostimulation of the hippocampal terminals. This is consistent with a recent study that showed a subset of RSC neurons located in superficial layers exhibited a large depolarization upon activation of hippocampal inputs (Yamawaki et al., 2019). In our tetrode recordings, we cannot determine the exact recording site for individual RSC neurons; however, there seems to be a bias toward recording more putative excitatory neurons in deep layers (layers 5 and 6) than that in superficial layers (layers 2 and 3), due to the larger size of excitatory neurons in deep rather than superficial layers. Nonetheless, the hippocampus seems to exert a net inhibition effect on the RSC, at least in deep layers of the RSC, which is intriguing. One possibility is that the hippocampus and RSC belong to two redundant systems, where the hippocampus system overshadows the RSC system in supporting certain learning and memory functions (Sutherland et al., 2010). When the hippocampus system is intact, it is the preferred functioning system that automatically suppresses RSC activity, which in turn facilitates hippocampal communication with other brain regions such as the entorhinal and prefrontal cortices. This possibility is supported by recent studies showing that impairment of both the hippocampus and RSC, but not impairment of either one disrupts learning abilities (Wiltgen et al., 2006; Coelho et al., 2018). Nonetheless, the hippocampus system seems to be the superior memory system since the RSC can only partially compensate hippocampal function in supporting the formation of short-term but not long-term memories (Zelikowsky et al., 2012). Another possibility is that hippocampal activity decreases RSC background activity to increase the signal-to-noise ratio of other inputs to the RSC, given that the RSC also receives extensive inputs from other brain regions, such as frontal cortices and thalamic nuclei (Vann et al., 2009).

### A RSC-to-Hippocampus Influence

In the current study, we used both ripple trough and ripple onset (Figure S2) as the reference to analyze ripple-correlated RSC activity, particularly the analysis of pre-ripple activation, which reached similar conclusions. This is expected because most ripple troughs lagged ripple onset, by only 30 ms or less (see STAR Methods), in contrast to the increased RSC activity starting much earlier, ~200 ms prior to the ripple (Figures 3 and S2). Nonetheless, the use of ripple trough is key to the detection of ripple phase locking of RSC putative inhibitory neurons, as using ripple onset in a similar analysis loses ripple phase information.

A similar pre-ripple activation has also been observed in several other cortical regions, including the entorhinal cortex, visual cortex, prefrontal cortex, and ACC (Isomura et al., 2006; Nir et al., 2011; Wang and Ikemoto, 2016; Ji and Wilson, 2007). Unlike many cortical excitatory neurons that display relatively low firing rates during SWS (<5 Hz), most RSC putative excitatory neurons displayed higher firing rates (Figure 2D), indicating a high active state of the RSC during SWS. Importantly, the pre-ripple activation of the RSC neurons depended on ripple amplitude (Figure 3C). These findings suggest that the RSC, and perhaps other cortices, likely play a role in influencing ripples, such as ripple-associated reactivation contents (Ji and Wilson, 2007; Rothschild et al., 2017), despite that the cortex itself is not required for ripple generation (Suzuki and Smith, 1988; Yamamoto and Tonegawa, 2017). Anatomically, the RSC does not project directly to the CA1. However, the RSC projects to several relevant brain regions, including the entorhinal cortex and subiculum, which both send direct outputs to the CA1 (Witter and Amaral, 1991; Sun et al., 2019). Together, communications from the RSC to CA1 is polysynaptic and may play an important role during the memory consolidation process.

Previous studies have shown that cortical neurons fire in clusters that are aligned with cortical Up states during SWS (Ji and Wilson, 2007; Wang and Ikemoto, 2016; Maingret et al., 2016). Similarly, we found that RSC population activity aggregated in clusters yet were separated by reoccurring silent periods of a few hundred milliseconds. The structure of these clusters seemed to be partially stereotypical because some RSC neurons fired at cluster initiation with an extremely high probability. The timing of individual RSC spikes in a cluster, whether to fire at the cluster start or end, seems to be relative rather than absolute, given that activity durations of RSC clusters, as well as the OFF-period durations, greatly vary across time. Nonetheless, this stereotypical pattern could represent the backbone of information coding units that are fundamental for communication with hippocampal ripples (Luczak et al., 2015). Further analysis on the exact timing and number of spikes within a cluster may reveal more detailed activity patterns that can convey specific information from the RSC to hippocampus, and vice versa. Together, these results provide evidence that RSC population activity, entrained by slow and fast oscillations, may influence hippocampal ripples and associated memory consolidation processes.

## STAR★METHODS

### LEAD CONTACT AND MATERIALS AVAILABILITY

Further information and requests for resources and reagents should be directed to and will be fulfilled by the Lead Contact, Dong V Wang (dw657@drexel.edu). This study did not generate new unique reagents.

### EXPERIMENTAL MODEL AND SUBJECT DETAILS

Male C57BL/6J mice were used in the current study: nine were used for dual-site *in vivo* recording, and six were used for optogenetics combined with *in vivo* recording (3–4 months old at the time of surgery; Jackson Laboratories). After surgery, mice were singly housed in cages (30 × 20 × 20 cm; referred to as home cage) with bedding, a water cup and a cardboard box filled with cotton pads (Wang et al., 2015) and kept on a 12 h light/dark cycle with *ad libitum* access to food and water. All procedures were approved by the Institutional Animal Care and Use Committee at Drexel University College of Medicine and were in accordance with National Research Council’s *Guide for the Care and Use of Laboratory Animals.*

### METHOD DETAILS

#### Stereotaxic Surgery

Similar surgery procedures were described in our previous publications (Wang et al., 2015; Wang and Ikemoto, 2016). Briefly, mice were anesthetized with a ketamine/xylazine mixture (~100/10 mg per kg, i.p.; Vedco Inc.). For dual-site recording, two bundles of eight tetrodes were implanted, one into the RSC and the other into the CA1, both in the right hemisphere. The coordinates for the RSC were AP −1.5 mm, ML 0.3 mm, and DV 0.9 mm; the coordinates for the CA1 were AP −2.2 mm, ML 1.7 mm, and DV 1.1 mm. For optogenetics combined with recording, pAAV1-CaMKIIa-hChR2 (H134R)-EYFP (0.2 μL; titer, ≥ 1 × 10^13^ vg/mL; Addgene #26969) was injected into the CA1 (AP −2.0 mm, ML 0.9 mm, and DV 1.3 mm), and an optic fiber (200 μm diameter; ThorLabs, Inc.) attached to eight tetrodes was implanted into the RSC (AP −1.5 mm, ML 0.3 mm, and DV 0.9 mm). Each electrode bundle was coupled with a microdrive (Wang et al., 2015) to allow gradual advancement of the electrodes into deeper brain regions post-surgery. For retrograde labeling, two cholera toxin subunit B tracers (CTB-555 and CTB-488 Alexa Fluor conjugate, Thermo Fisher Scientific; 0.15 μL; 100 mg/ml) were injected into the RSC of the left and right hemispheres, respectively. Mice were sacrificed for imaging processes after one week.

#### Tetrode Recording

We used tetrodes for recording, uniform to our previously publications (Wang et al., 2015; Wang and Ikemoto, 2016). Each tetrode consisted of four wires (90% platinum and 10% iridium; 18 mm diameter with an impedance of ~1−2 MΩ for each wire; California Fine Wire). About three days after surgery, tetrodes were screened for neural activity. All of the analyzed data were recorded at least one week after surgery. Neural signals were preamplified, digitized, and recorded using a Neuralynx Digital Lynx acquisition system; the animals’ behaviors were simultaneously recorded. Each electrode bundle was lowered by 50−100 μm daily until clear CA1 ripples or activity of multiple RSC neurons were detected. Local field potentials (LFPs) were digitized at 2 kHz and filtered at 1−500 Hz, whereas spikes were digitized at 30 kHz and filtered at 600−6000 Hz, using ground as the reference for both. All mice received 2–5 sessions of recording when they were freely-behaving or sleeping in home cages (2–4 h). After completion of the above recording sessions, the RSC electrode bundle was lowered by 50−100 μm to record a deeper site in the RSC, while the CA1 electrode bundle stayed in the same location if large-amplitude ripples (peak amplitude exceeding 6 standard deviations) were recorded. A total of 2–5 sites in the ventral portion of RSC were recorded from each mouse. Before optogenetic stimulation experiments, we waited three weeks or longer after surgery to allow expression of ChR2 on hippocampal terminals. Three parameters of stimulation (one, three and five pulses of laser stimulation; 25 Hz; pulse width, 3 ms; laser, ~1 mW) were used in each session. Pulses of blue laser (473 nm; Opto Engine LLC) were delivered intermittently with an intertrial interval of 10–15 s when mice were freely-behaving or sleeping in home cages (1–2 h). Data from the three and five pulses of stimulation were combined for analyses as shown in Figures 4 and 5.

#### Histology

Mice were deeply anesthetized, and the final electrode position was marked by passing a 10 μA, 20 s current through two tetrodes. Then, mice were intracardially perfused with PBS followed by 4% paraformaldehyde (PFA) or 10% formalin. Brains were removed and post-fixed in the PFA or formalin, allowing for ≥ 24 h before slicing on a vibratome (50 mm coronal sections; Leica). Sections were mounted with a Mowiol mounting medium (Mowiol 4–88, Calbiochem) based on a recipe from Cold Spring Harbor Protocols (pdb.rec10255).

### QUANTIFICATION AND STATISTICAL ANALYSIS

The detection of rapid-eye movement (REM) and slow-wave sleep (SWS) has been described before (Wang et al., 2015). Briefly, it was determined by the theta (6−10 Hz)/delta (1−4 Hz) ratio extracted from the power spectra of hippocampal CA1 LFPs when mice remained immobile in their home cage’s ‘bed’ - a cardboard box filled with cotton material, where they exclusively slept. A ratio of 2 or greater was identified as REM, whereas a ratio of 1 or lower was identified as SWS. We used Plexon Offline Sorter for spike sorting, and sorted spikes were further analyzed in NeuroExplorer (Nex Technologies) and MATLAB (Mathworks). The awake immobility was determined predominantly when the mice were consuming rodent chow or rice. (At other awake times, mice were typically active with extremely sparse CA1 ripple occurrence).

Analyses on RSC spikes were based on a sample size of 170 RSC neurons recorded from nine dual-site recording mice (39, 38, 29, 17, 11, 11, 10, 8, and 7 neurons from each mouse) and another 102 RSC neurons recorded from six optostimulation mice ( 34, 26, 19, 18, 3, and 2 neurons from each mouse). This sample size is comparable to previous RSC recordings (Alexander and Nitz, 2015; Alexander et al., 2018; Koike et al., 2017) despite being relatively small (mainly because the high frequency firing of RSC neurons hindered spike sorting; therefore, many not-so-well isolated RSC units were excluded from further analysis). Additionally, low-frequency neurons (< 0.5 Hz) were excluded from analyses due to an insufficient number of spikes.

RSC slow waves were bandpass filtered at 1−4 Hz (MATLAB), and an Up (or Down) state was defined as the period when an amplitude of the filtered LFP was> 1 SD above (or < 1 SD below) mean and lasted > 50 ms (Wang and Ikemoto, 2016). CA1 ripples and RSC ripple-like oscillations were bandpass filtered between 150−50 Hz using a zero-phase filter in NeuroExplorer (IIR Butterworth; filter order = 5). For ripple onset detection, the ripple envelope was computed using a Hilbert transform and smoothed with a Gaussian filter (4 ms stdev) (Karlsson and Frank, 2009). Ripple onset was defined as the onset of the smoothed ripple envelope exceeding 3 standard deviations above the mean (Figure S2) that lasted 15 ms or longer (Karlsson and Frank, 2009). The definitions of ripple trough and ripple size were similar to that reported in our earlier publication (Wang and Ikemoto, 2016). Briefly, high-amplitude ripples have a peak amplitude exceeding 8 standard deviations (SD) below the mean, while middle- and low-amplitude ripples have peak amplitudes exceeding 6 and 4 SDs below the mean, respectively. High-amplitude plus middle-amplitude ripples were used for all the analyses except that shown in Figure 3C, where different sizes of ripples were used separately for analyses. To give an idea about the time lag between ripple onset and ripple trough, ~86% of ripple troughs lagged ripple onsets by 5−30 ms in our analyses.

Z-score transform was calculated as: Zi = (Xi − x) / σ; where Zi and Xi were z-scored and actual values of individual data points, respectively, while x and σ were mean and standard deviation (SD), respectively, derived from baseline data points (see legends of Figures 1, 2, 3, and 5). Briefly, for z-score transform of the spectrogram, peri-ripple spectrograms were first averaged across several recordings (2–3 sessions) for individual animals, and then averaged across animals (n = 9). Mean and SD were calculated between −1.0 and −0.5 s for individual averaged frequency bands of the spectrogram. For z-score transform of the correlogram between ripple and RSC spikes (bin size = 10 ms), mean and SD were calculated between −1.0 and −0.5 s, and a neuron was considered activated if the post-ripple z-score (0−10 ms) exceeded 3.3 (p < 0.001). For z-score transform of the correlogram between optostimulation and RSC spikes (bin size = 0.5 ms), mean and SD were calculated between −60−0 ms: a neuron was considered activated if the post-stimulation peak z-score (averaged across the three pulses of optostimulation; see Figure 5) exceeded 3.3 within 10 ms, while a neuron was considered inhibited if the post-stimulation mean z-score (averaged across the three pulses of optostimulation between 0−20 ms) was below −3.3. SD, standard deviation; SEM, standard error of the mean.

PCA analysis was conducted in MATLAB (Sadacca et al., 2016), based on normalized spike waveforms between 0−1 ms (Figure 2A). We first extracted the three major principal components (PC1, PC2 and PC3), and then used a hierarchical clustering algorithm (Linkage) to find the similarity (Euclidean distance) between all pairs of spike waveforms in principal components space, iteratively grouping the spike waveforms into larger and larger clusters based on their similarity. Lastly, we set a distance-criterion to extract three clusters from the hierarchical tree (Figure 2C).

### DATA AND CODE AVAILABILITY

The data and code supporting the current study are available from the corresponding author on request.

## Supplementary Material

1

2

## Figures and Tables

**KEY RESOURCES TABLE:** 

REAGENT or RESOURCE	SOURCE	IDENTIFIER
Bacterial and Virus Strains		
pAAV-CaMKIIa-hChR2(H134R)-EYFP	Lee et al., 2010	Addgene #26969
Chemicals, Peptides, and Recombinant Proteins		
Cholera Toxin Subunit B (Recombinant), Alexa Fluor 555 Conjugate	ThermoScientific/ Invitrogen	Cat#C22843
Cholera Toxin Subunit B (Recombinant), Alexa Fluor 488 Conjugate	ThermoScientific/ Invitrogen	Cat#C34775
Mowiol 4-88	Calbiochem	Cat#9002-89-5
Deposited Data		
Raw and analyzed data	This paper	Available upon request
MATLAB code	This paper	Available upon request
Experimental Models: Organisms/Strains		
Mouse: C57BL/6J	The Jackson Laboratory	JAX: 000664
Software and Algorithms		
Adobe Photoshop CS6	Adobe Systems	N/A
Adobe Illustrator CS6	Adobe Systems	N/A
MATLAB	MathWorks	N/A
NeuroExplorer v4	Nex Technologies	N/A
Offline Sorter v4	Plexon Inc	N/A
Other		
Stereotaxic frame	Kopf Instruments	N/A
Microinjection pump	WPI	Cat#UMP3T-2
Optic fiber (200-μm diameter)	ThorLabs, Inc.	Cat#FT200EMT
Tetrode Wire	Califonia Fine Wire Co.	Cat#CFW0013809
Digital Lynx acquisition system	Neuralynx	N/A
473 nm laser	Opto Engine LLC	Cat#MBL-III-473
Vibratome	Leica Biosystems Inc.	Cat#VT1000S

## Highlights

- Both the hippocampus and RSC play indispensable roles in memory formation
- The RSC displays a pre-ripple activation and fast oscillation
- Hippocampal ripple activates RSC interneurons and inhibits RSC principal neurons
- The hippocampus may overshadow the RSC in supporting memory functions
