# Supplementary material for: Hippocampal Ripple Coordinates Retrosplenial Inhibitory Neurons during Slow-Wave Sleep
Source: Cell Rep. Author manuscript; Available in PMC 2020 Feb 9. (PMC7007963; doi:10.1016/j.celrep.2019.12.038)
Supplement: 1 [file NIHMS1549889-supplement-1.pdf]

Cell Reports, Volume *30*

Supplemental Information

Hippocampal Ripple Coordinates
Retrosplenial Inhibitory Neurons
during Slow-Wave Sleep

Ashley N. Opalka, Wen-qiang Huang, Jun Liu, Hualou Liang, and Dong V. Wang

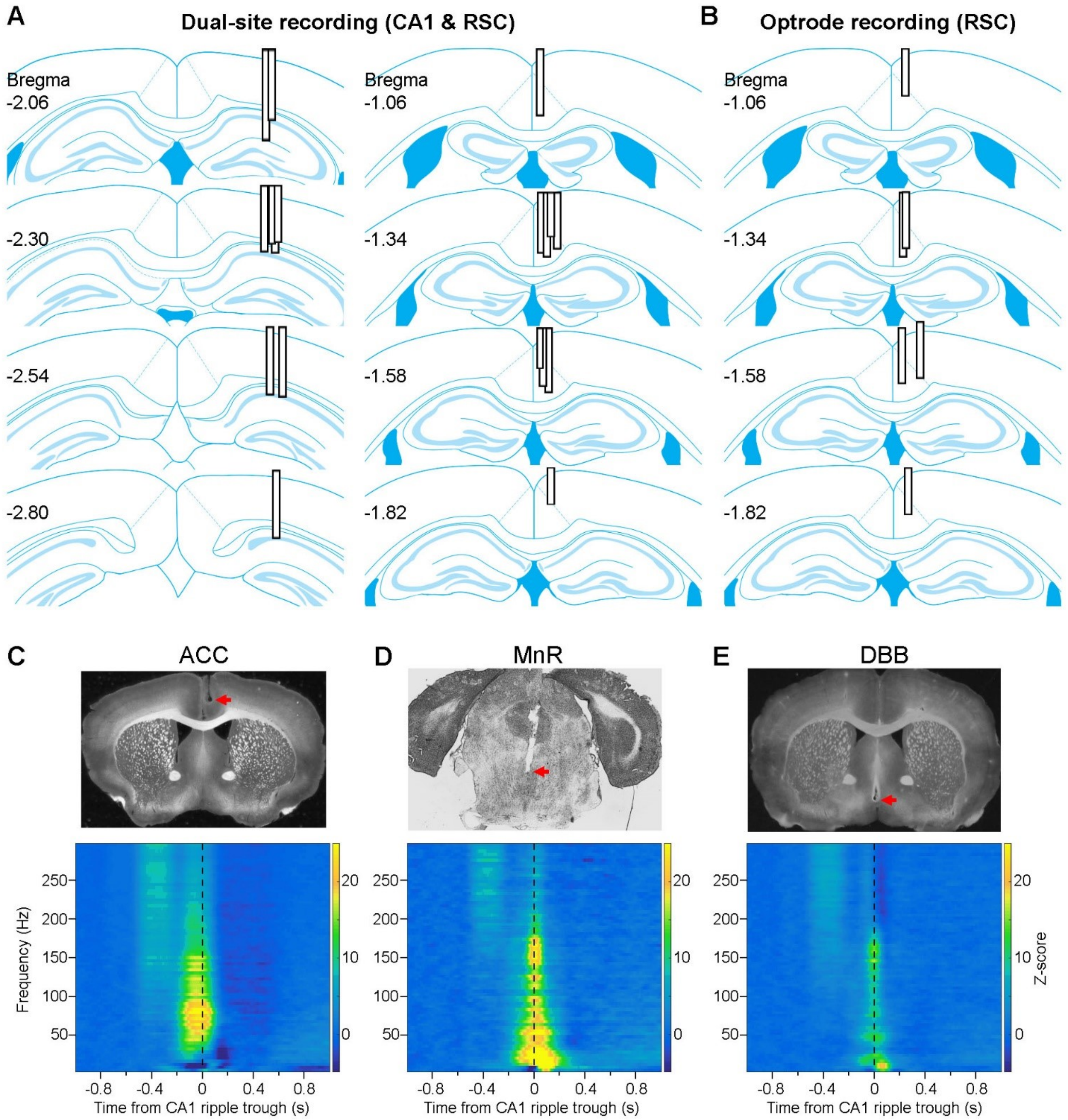

Figure S1. Final recording sites and peri-ripple spectrograms (related to Figures 1&4).
(A) Schematics of the recording sites for dual-site recording mice (n = 9). Adapted from Franklin & Paxinos 2008.
(B) Schematics of the recording sites for optostimulation mice (n = 6).
(C–E) Top, coronal brain section showing the recording site (red arrow) in the anterior cingulate cortex (C; ACC), median raphe (D; MnR), and diagonal band of Broca (E; DBB). Bottom, mean peri-ripple spectrogram of ACC (C), MnR (D), and DBB (E) LFPs in relation to CA1 ripple trough (n = 3 mice for each site).

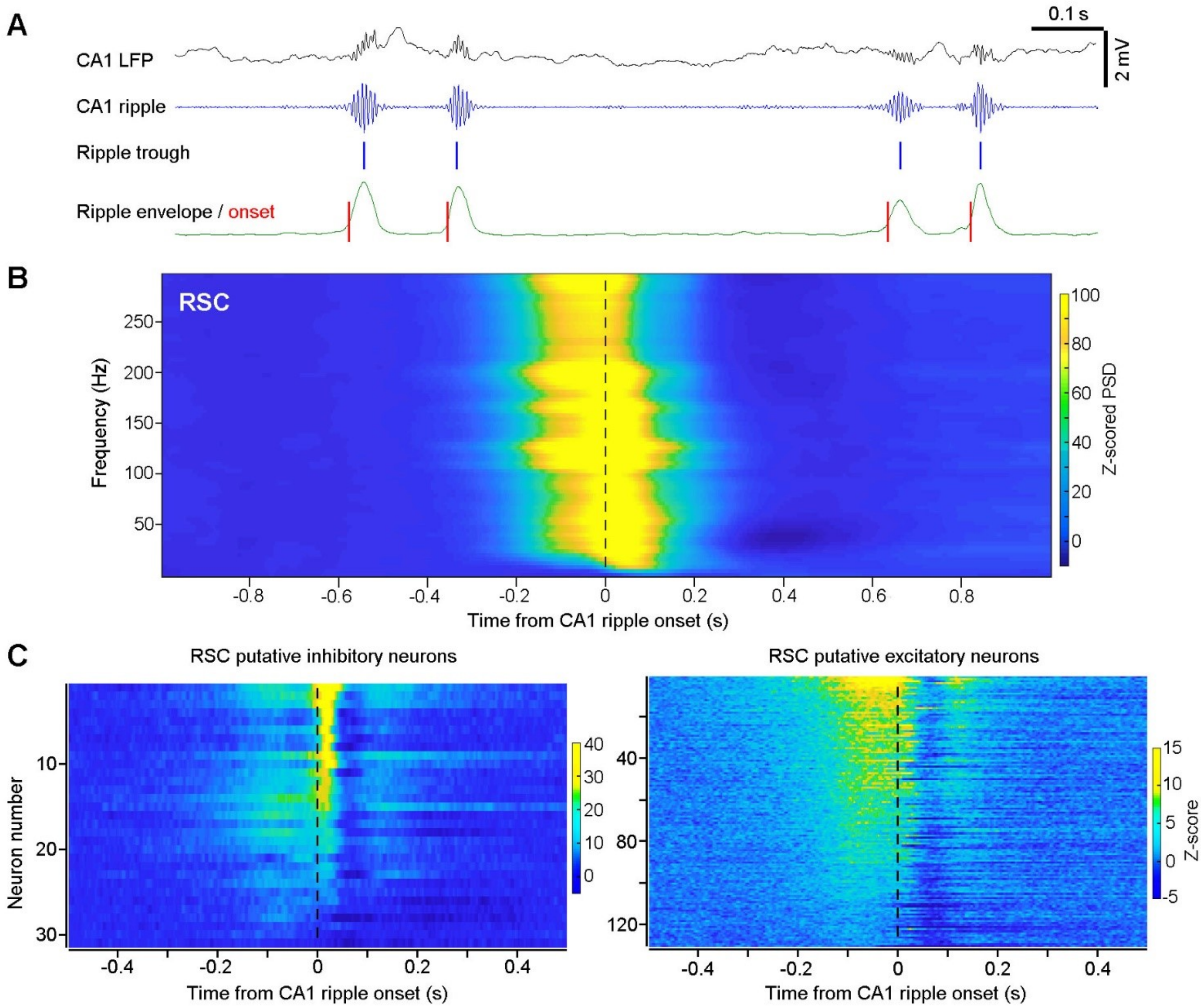

Figure S2. Ripple onset analyses of RSC oscillations and spikes (related to Figures 1&3).
(A) Representative CA1 LFP (1–500 Hz, black), filtered ripple (150–250 Hz, blue), identified ripple trough (blue bars), ripple envelope (green; see Methods) and identified ripple onset (red bars).
(B) Mean peri-ripple spectrogram of RSC LFPs in relation to CA1 ripple onset (n = 9 mice). PSD, power spectral density. Z-score transform was based on mean and SD calculated between -1 and -0.5 s.
(C) Heatmap activity of RSC putative inhibitory (left; n = 31) and excitatory neurons (right; n = 130) in relation to ripple onset. Z-score transform was based on mean and SD calculated between -1 and -0.5 s.

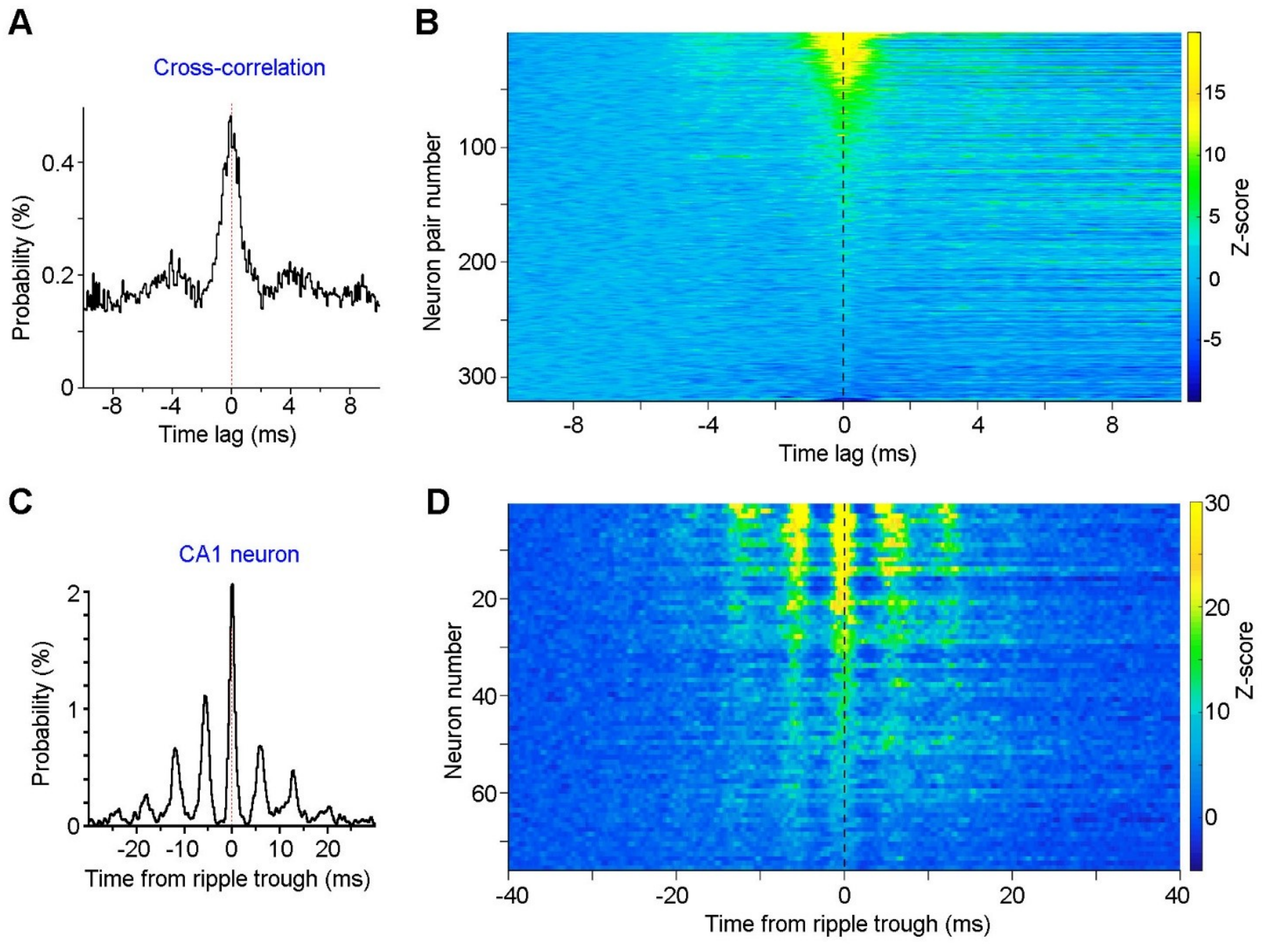

Figure S3. RSC and CA1 putative excitatory neurons (related to Figures 2&3).
(A) Cross-correlation histogram of two RSC putative excitatory neurons, indicating synchronized firing (±1 ms) of the two neurons. Bin, 0.1 ms.
(B) Cross-correlation heatmap of RSC excitatory neuron pairs (n = 320 pairs) that were recorded simultaneously from different tetrodes. Z-score transform was based on mean and SD calculated between -10 and -5 ms.
(C) Firing probability of a CA1 putative pyramidal neuron in relation to CA1 ripple trough. Bin, 0.5 ms.
(D) Activity heat map of 76 CA1 putative pyramidal neurons in relation to ripple trough. Z-score transform was based on mean and SD calculated between -40 and -20 ms.

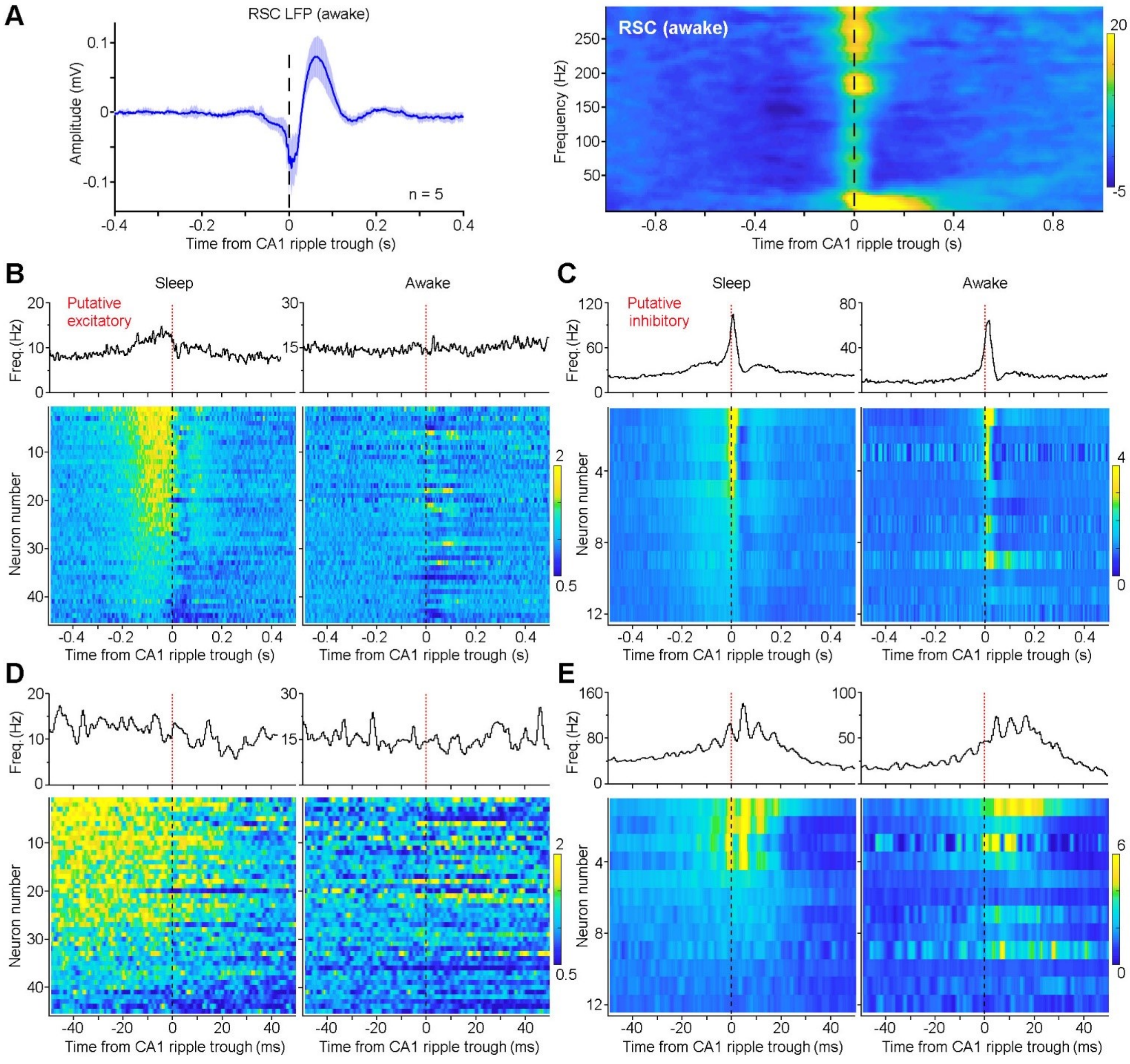

Figure S4. Ripple correlated RSC activity during sleep and awake immobility (related to Figure 3).
(A) Mean ± SD of RSC LFPs (left) and mean peri-ripple spectrogram of RSC LFPs (right) during awake immobility in relation to CA1 ripple trough (n = 5 mice). Color bar, z-scored PSD.
(B&C) Representative (top panels) and individual (bottom panels) RSC putative excitatory (B; n= 45) and inhibitory neurons (C; n = 12) in relation to ripple during sleep (left panels) and awake immobility (right panels). Bin, 2 ms.
(D&E) Zoomed-in view of B and C (within ±50 ms), respectively. Bin, 0.5 ms.
Color bars in B–D represent normalized firing frequency (divided by mean frequency calculated between -1 and -0.5 s). Note that the pre-ripple activation (-0.2–0 s) of both putative excitatory (B) and inhibitory (C) neurons is absent during awake immobility. A subset of putative inhibitory neurons displays a ripple-phase locked firing during both SWS and awake immobility (E).

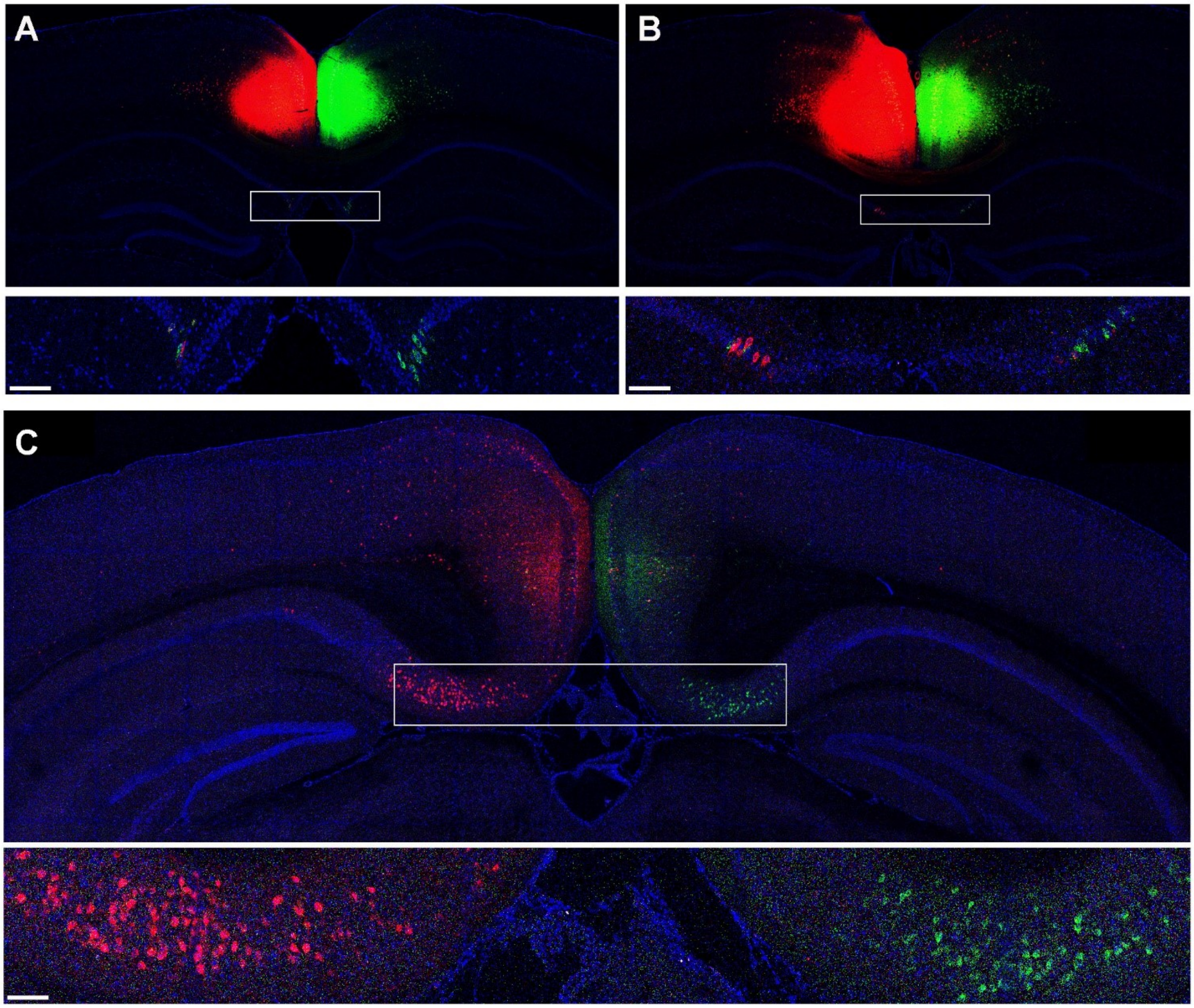

Figure S5. Retrograde labeling of RSC inputs (related to Figure 4).
(A&B) The RSC was injected with the retrograde tracer CTB-555 (left hemisphere; 0.15 μL) and CTB-488 (right hemisphere; 0.15 μL). Inset, CTB labeling in the fasciola cinereal (A) and rostral medial CA1 (B).
(C) Dense CTB labeling in the dorsal subiculum.
Sections in A–C are from the same brain. Scale bars, 0.1 mm.

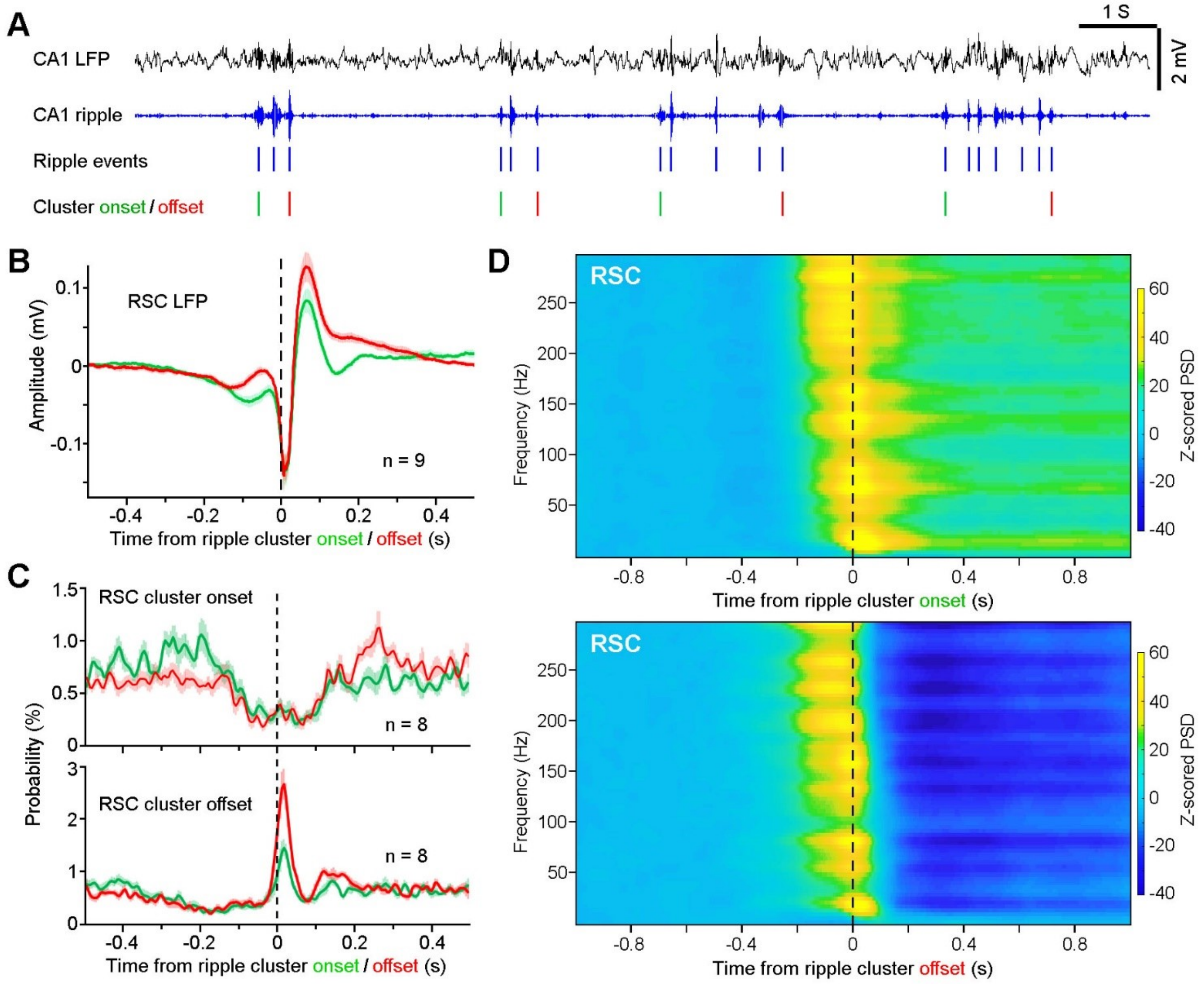

Figure S6. CA1 ripple cluster analysis (related to Figures 1&6).

(A) Representative CA1 LFP (1–500 Hz, black), filtered ripple (150–250 Hz, blue), identified ripple events (blue bars), and identified ripple onset/offsets (green and red bars, respectively). Ripple cluster was defined as three or more consecutive ripple events occurring above 1 Hz.

(B) Mean amplitude ± SEM of RSC LFPs (n = 9 mice) in relation to CA1 ripple cluster onsets (green) and offsets (red). Bin, 1 ms.

(C) Mean probability ± SEM of RSC cluster onsets (top; n = 8 sessions) and RSC cluster offsets (bottom; n = 8 sessions) in relation to CA1 ripple cluster onsets (green) and offsets (red). Bin, 5 ms.

(D) Mean peri-ripple spectrogram of RSC LFPs (n = 9 mice) in relation to CA1 ripple cluster onsets (top) and offsets (bottom). Bin, 5 ms.
